# Immobilization of Chondroitin Sulfate A onto Monolithic Epoxy Silica Column as a New Chiral Stationary Phase for High-Performance Liquid Chromatographic Enantioseparation

**DOI:** 10.3390/ph14020098

**Published:** 2021-01-27

**Authors:** Ratih Ratih, Hermann Wätzig, Azminah Azminah, Mufarreh Asmari, Benjamin Peters, Sami El Deeb

**Affiliations:** 1 Institute of Medicinal and Pharmaceutical Chemistry, Technische Universität Braunschweig, 38106 Braunschweig, Germany or ratih_rath@staff.ubaya.ac.id (R.R.); h.waetzig@tu-braunschweig.de (H.W.); 2 Department of Pharmaceutical Chemistry, Faculty of Pharmacy, University of Surabaya, Surabaya 60284, Indonesia; azminah@staff.ubaya.ac.id; 3 College of Pharmacy, King Khalid University, Abha 62529, Saudi Arabia; masmri@kku.edu.sa; 4 Instrumental Analytic R&D, Merck KGaA, 64293 Darmstadt, Germany; benjamin.peters@merckgroup.com

**Keywords:** amlodipine, chiral stationary phase, chondroitin sulfate A, enantioseparation, immobilization, monolithic column, Schiff base, verapamil

## Abstract

Chondroitin sulfate A was covalently immobilized onto a monolithic silica epoxy column involving a Schiff base formation in the presence of ethylenediamine as a spacer and evaluated in terms of its selectivity in enantioseparation. The obtained column was utilized as a chiral stationary phase in enantioseparation of amlodipine and verapamil using a mobile phase consisting of 50 mM phosphate buffer pH 3.5 and UV detection. Sample dilution by organic solvents (preferably 25% *v*/*v* acetonitrile-aqueous solution) was applied to achieve baseline enantioresolution (*R*s > 3.0) of the individual drug models within 7 min, an excellent linearity (*R*^2^ = 0.999) and an interday repeatability of 1.1% to 1.8% RSD. The performance of the immobilized column for quantification of racemate in commercial tablets showed a recovery of 86–98% from tablet matrices. Computational modeling by molecular docking was employed to investigate the feasible complexes between enantiomers and the chiral selector.

## 1. Introduction

Polysaccharide-based chiral stationary phases (CSPs) play an important role in enantioseparations of chiral compounds by high-performance liquid chromatography (HPLC) [1]. Due to the asymmetric and long-range helical structures, polysaccharides offer high recognition capacity and enantioselectivity toward broad types of chiral substances [2,3]. As one of the most prominent separation methods in analysis and preparative purposes, HPLC using amylose-based and cellulose-based columns delivers excellent performance [4,5,6,7]. In the beginning, the utilization of polysaccharide-based CSPs faced a restriction in the enantioselectivity improvement due to their low compatibility toward polar organic modifiers [8,9]. Therefore, immobilized CSPs are developed to achieve an expansion of column compatibility with a wide range of solvent polarity [9,10]. Immobilized CSPs typically could be applied in normal phase (NP)-, reversed-phase (RP)-, and polar-elution mode with a large diversity of organic solvents as mobile phases [1,11]. On the other hand, coated CSPs can only be used as a single mode in NP or RP.

Immobilization of a chiral selector onto macroporous silica has been conducted through a radical copolymerization reaction [12] and a photochemical technique [9]. In 2017, Bezhitashvili et al. reported highly efficient separation using a wide pore silica surface as the backbone for high molecular weight polysaccharide-based chiral selectors [12,13] by providing loading capacity [14]. In line with these achievements, a previous study on the covalent immobilization of monolithic silica via epoxide moiety has improved separation efficiency and compatibility with numerous organic solvents [15].

**Figure 1:**
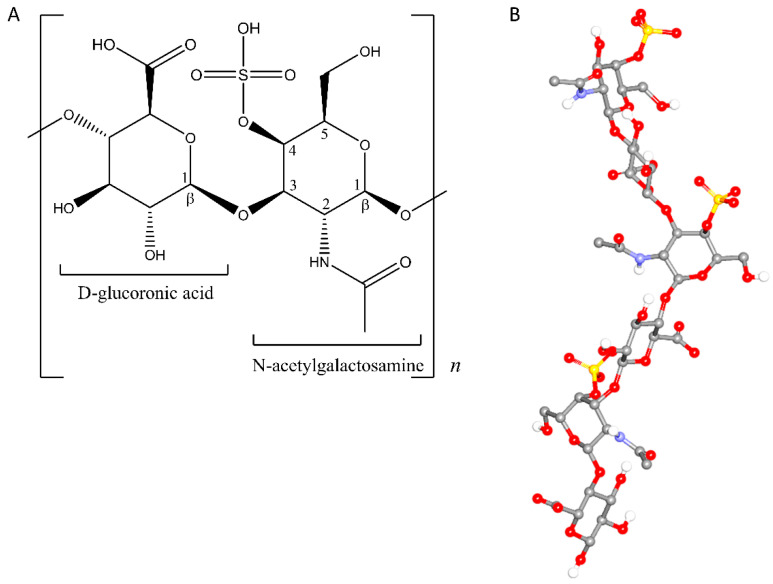
Repeating unit of chondroitin sulfate A (CSA): 2-D monomer (**A**) and 3-D helical representation consisting of three monomer units, extracted from its crystal structure entry 4N8W [20] (**B**).

Among polysaccharides, chondroitin sulfates have been found to be potential chiral selectors in capillary electrophoresis (CE) [16]. As charged molecules, they possess different mobility under an electrical field compared to neutral polysaccharides. In addition to their charges, chondroitin sulfates afford ionic and hydrophobic interactions as well as hydrogen bonds. Therefore, chondroitin sulfate A (CSA) with the sulfate group at carbon number four (Figure 1) has been successfully utilized as a chiral selector in CE [17]. Since then, chondroitin sulfate B [18], D, and E [19] have been employed as chiral selectors for various chiral substances.

Monolithic silica columns showed successful applications as one current approach for fast HPLC analysis [3,21,22,23]. A combination of the broad enantiomers recognition ability of CSA and the benefits of the monolith backbone is interesting for further investigation of chromatographic enantioseparations. The immobilization of a chiral selector on a monolithic chromatographic support through epoxy and Schiff base formations have been established [24,25,26,27]. Particularly, its application on monolithic epoxy support in capillary liquid chromatography (CLC) [28] and HPLC via in situ modification has been successfully obtained [15,27]. Moreover, in general, immobilization through a Schiff base formation proves to be the best approach [24]. Based on our best knowledge, the usage of CSA as a chiral selector in HPLC either as a mobile phase additive or a CSP has not been reported.

Molecular docking is commonly used in chiral separation studies to clarify the chiral recognition mechanism [29,30]. A docking approach might be employed to simulate the interactions between a chiral selector and the individual enantiomers. The most stable intermolecular conformation could be simulated with regard to the lowest binding energy [30].

In this study, the immobilization of CSA onto a monolithic column by sequential Schiff base formation and reduction was developed to provide an alternative polysaccharide-based CSP for chromatographic enantioseparation. The enantioseparation capability of the immobilized CSA column was evaluated by using amlodipine (AML) and verapamil (VER) in their racemic form as chiral analytes. Both compounds are calcium channel antagonist drugs, belonging to the classes of dihydropyridines and non-dihydropyridines, respectively. Further evaluation of the column performance in enantioseparation was carried out using commercial tablet matrices. Additionally, molecular docking was utilized to simulate the enantiorecognition and visualize the feasible interaction between CSA and the individual enantiomers.

## 2. Results

### 2.1. Organic Modifier Effects

The polarity of the mobile phase can be tuned by changing the type and concentration of an organic additive. Consequently, the recognition mechanism can be strongly influenced [31,32,33]. The organic solvent can be added in the mobile phase or as sample diluent. In order to enhance the enantioresolution of amlodipine (AML) and verapamil (VER), the addition of an organic modifier in the sample solvent was applied. The concentrations of methanol (MeOH) and acetonitrile (MeCN) in aqueous solution as sample solvents were optimized, while the mobile phase composition was kept constant. The effect of organic modifier content on enantioseparation was investigated at 10–100% *v*/*v* MeOH (in water) and 10–100% *v*/*v* MeCN (in water).

**Figure 2:**
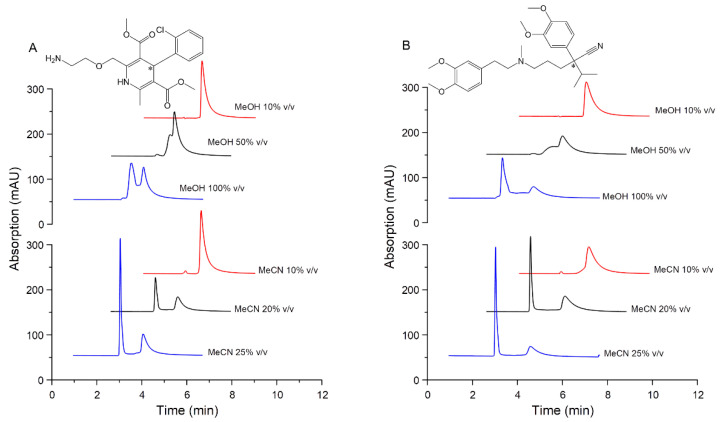
Influence of methanol (MeOH) and acetonitrile (MeCN) as organic solvents in various concentrations of sample diluent towards enantioseparation profiles of (**A**) amlodipine, AML and (**B**) verapamil, VER, as racemic mixtures. Stationary phase: CSA column; mobile phase: 50 mM phosphate buffer pH 3.5; flow rate 0.8 mL/min; UV detection (**A**) 240 nm and (**B**) 230 nm. The organic solvent was prepared in water (aqueous solution).

The increases of MeOH concentrations in the sample solvent gradually improved the resolution (*R*_s_) of AML and VER. However, up to the solvent composition of 100% *v*/*v* MeOH, only partial enantioseparations of AML and VER were reached with *R*_s_ = 0.8 and *R*_s_ = 1.1, respectively. The appearances of peak plateaus and coalescence indicate competition between chromatographic resolution and on-column stereoisomerism [34], known as dynamic interconversion HPLC [35]. The racemization of the analyte during passing throughout the column drives the rise of the baseline pattern [36]. In some cases, low-temperature conditions in chromatographic separations were applied to prevent interconversion by conducting a slow exchange system [34,35]. In this study, the addition of 10% *v*/*v* to 25% *v*/*v* MeCN (in water) as a sample solvent significantly increased the response of the first eluted enantiomer and led to complete peak decoalescence. The influence of organic modifiers as the sample diluent toward enantiomeric separation profiles is shown in Figure 2.

### 2.2. Resolution and Selectivity

Resolution (*R*_s_) of AML and VER was obtained at *R*_s_ = 3.2 and *R*_s_ = 3.6, respectively. Furthermore, the enantioselectivity of *α* = 1.8 for AML and *α* = 2.1 for VER, was achieved as shown in Table 1.

### 2.3. Method Validation

Proportional responses of the analyte in triplicate injections of five concentrations have shown decent linearity for both drug models. Intraday and interday repeatabilities of peak area ratio were obtained at 0.5–1.3% and 1.1–1.8% RSD, respectively. The RSD values are indicating satisfactory precision of the separation method. Method accuracy by adding a respective amount of analyte at 50 µg/mL, 80 µg/mL, and 100 µg/mL was achieved at 98–102% and 95–104% for AML and VER, respectively. Validation parameters are shown in Table 2.

### 2.4. Enantioseparation in Commercial Tablet Matrices

**Figure 3:**
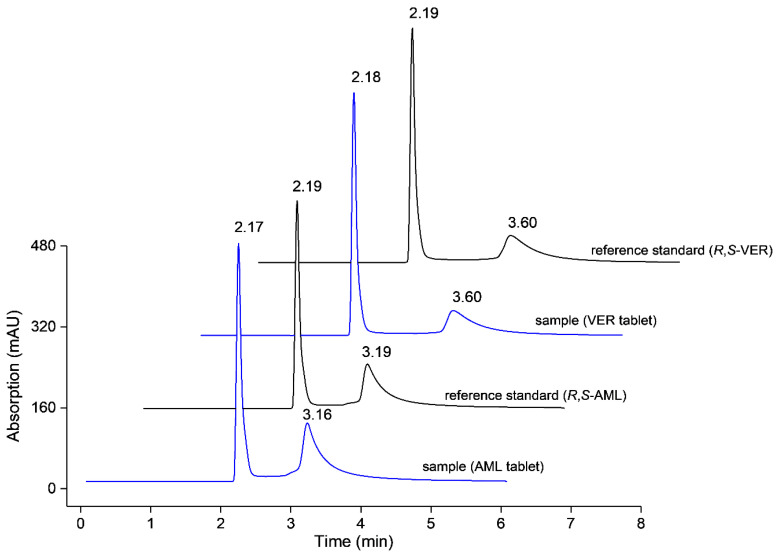
Determination of active pharmaceutical ingredients in commercial tablet matrices. The chromatograms represent amlodipine (AML) 80 µg/mL and verapamil (VER) 100 µg/mL with their individual reference standards at the corresponding concentrations.

The modified column also proved its selectivity towards commercial tablets’ matrices of A (5 mg AML) and B (80 mg VER). Sample pretreatment through solvent addition (25% *v*/*v* MeOH in water) and ultrasonication was taken to release the analyte from its matrix and followed by filtration. The proposed method was able to determine the active substance of selected pharmaceutical dosage form in triplicate with an average of 86% (2.5% RSD*, n* = 9, *df* = 6) and 98% (3.0% RSD, *n* = 9, *df* = 6) for AML and VER, respectively. Separation profile of the enantiomers in tablet matrices is given in Figure 3.

### 2.5. Intermolecular Interactions Revealed by Molecular Docking

As per molecular docking, the interactions between CSA and enantiomers seem to be driven by hydrogen bonding. The interactions of CSA towards *R*-(+)-amlodipine were dominated by hydrogen bonding between (SO···) and (···HN) of the primary amine, among two electrostatic binding forces. In contrast, other than a conventional hydrogen bond and an electrostatic interaction, the primary amine of *S*-(−)-amlodipine possessed electrostatic π-anion (chlorophenyl ring··· and ···O-S) interaction, which were not found in its antipode. The different types of interactions, along with the resulted binding affinities, may lead to the enantioseparation of AML.

**Figure 4:**
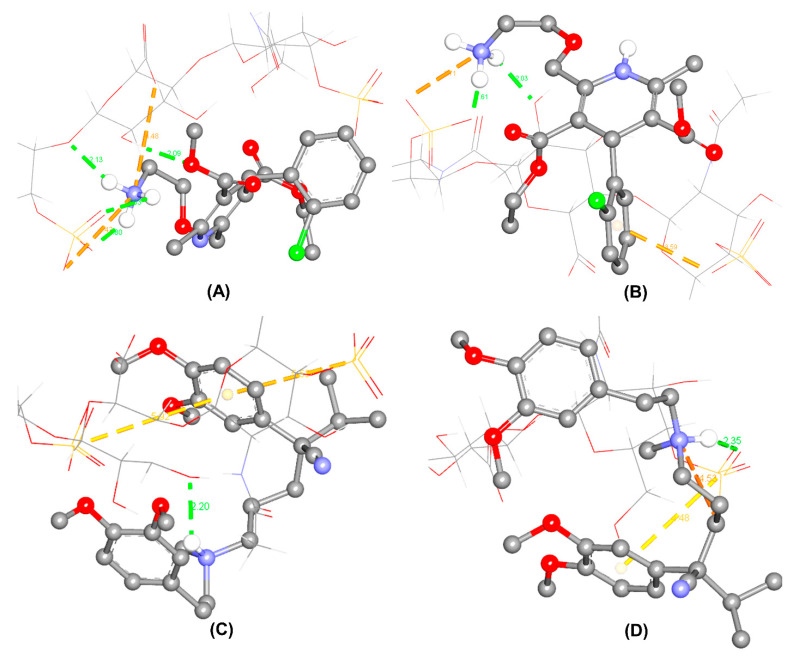
Prediction of the most stable complexes between CSA and the enantiomers of amlodipine and verapamil. CSA is drawn in the wire form and enantiomers are illustrated in the ball-stick form as *R*-(+)-amlodipine (**A**); *S*-(−)-amlodipine (**B**); *R*-(+)-verapamil (**C**); *S*-(−)-verapamil (**D**). Green dotted lines represent hydrogen bonds; yellow and orange dotted lines represent π-sulfur interactions and electrostatic interactions, respectively. The numbers indicate the distances between two functional groups in the predicted interactions.

In the case of VER, the secondary amine plays an important role in the molecular interaction. *R*-(+)-verapamil formed a hydrogen bond from the interaction between the ionized tertiary amine and (···-O-) group of CSA. On the other hand, *S*-(−)-verapamil formed an hydrogen bond from the interaction between the ionized tertiary amine (NH···) and (···O=S) group of CSA. Two π-sulfur interactions were found in the *R*-(+)-enantiomer and only one in the *S*-(−)-enantiomer. Additionally, an electrostatic interaction was only present in by *S*-(−)-verapamil. Thus, the differences in the complexes of VER enantiomers might occur. The populations in the best clusters from 250 docking runs for each enantiomer were 44 for *R*-(+)-amlodipine, 67 for *S*-(−)-amlodipine, 28 for *R*-(+)-verapamil, and 19 for *S*-(−)-verapamil. The estimated lowest binding energies in the best clusters are listed in Table 3, which correspond to the complexes and the molecular interactions briefly depicted in Figure 4.

## 3. Discussion

### 3.1. Immobilized CSA Column

**Figure 5:**
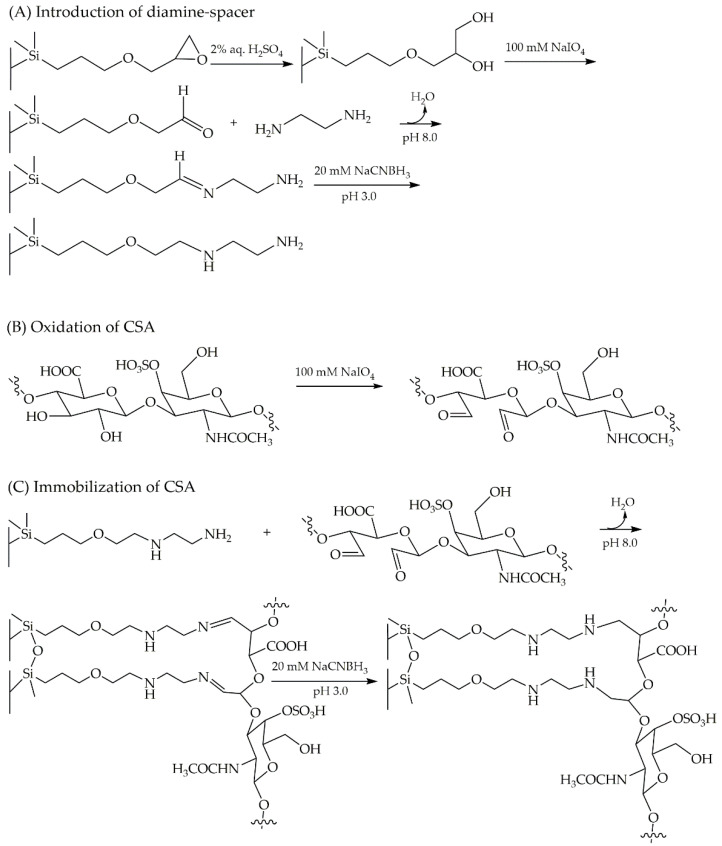
Schematic immobilization of CSA onto the monolithic epoxy column. Introduction of diamine-spacer (**A**), oxidation of chondroitin sulfate A (CSA) (**B**), and immobilization of CSA (**C**).

A protocol of stepwise immobilization onto monolithic epoxy HPLC column via reductive amination has been proposed by Merck. This approach is applicable for immobilization of various ligands, including performing in house generation of CSA-based CSP. The strategy of CSA immobilization onto the monolithic epoxy column was initialized by opening the epoxy ring through hydrolyzation to a diol. The oxidizing agent NaIO_4_ was utilized to cleave the diol groups to an aldehyde. The formation of aldehydes was intended to provide an active surface for the attachment of diamine-spacer. The primary amines of the spacer were expected to bind to aldehydes by forming a Schiff base linkage (imine formation) under alkaline conditions. Ethylenediamine, as the selected diamine-spacer, was introduced into the column in a continuous flow system with a reductant (NaCNBH_3_, 5 mM) under mildly basic conditions ((NH_4_)_2_SO_4_, 1.9 M) at pH 8.0. In the next step, NaCNBH_3_ (20 mM) was utilized at pH 3.0 to drive the immobilization of ethylenediamine to completion by quenching the remaining residual aldehydes on the monolithic surface. In order to neutralize the immobilized column, a phosphate buffer was employed at pH 7.4 (Figure 5A). A solution of 100 mM NaIO_4_ in 20% *v*/*v* MeOH (aqueous solution) was prepared to conduct the oxidative cleavage of CSA, prior to introducing to the column (Figure 5B). The immobilization of CSA (Figure 5C) was carried out via Schiff base formation using reductant NaCNBH_3_ (5 mM) under a mild basic condition of (NH_4_)_2_SO_4_ (1.9 M) pH 8.0 in a continuous flow system. A subsequent process of quenching the remaining aldehydes employing NaCNBH_3_ (20 mM) at pH 3.0 and column neutralization using phosphate buffer pH 7.4 led to the completion of CSA immobilization.

In this protocol, the primary amine as a spacer was expected to bind to aldehydes via Schiff base linkage. However, the Schiff base linkage is susceptible to hydrolysis and can revert to the aldehyde and primary amine. Thus, the linkage needs to be stabilized by reduction to a secondary amine bond. Therefore, the reaction was conducted using reductant NaCNBH_3_ (5 mM) to perform an in situ reductive amination reaction. The reaction between diamine groups and aldehydes might also give side products such as imidazolines, formed as aminal from the diamine, or diimines from reactions of neighboring aldehydes of the stationary phase [37]. Nevertheless, the immobilization process of ethylenediamine in this scheme was focused on the formation of imines as the main product.

In order to conduct applicability and simplicity, the immobilization was performed on a monolithic analytical column. The immobilization was carried out in a closed continuous flow system to carry out a constant and stable process. In the applied protocol, an in-process determination of yields and column characterization was not possible. Therefore, subsequent rinsing was employed using NaIO_4_ at an alkaline pH as a control strategy to minimize unwanted side products and stabilize the intended reaction. However, the presence of NaIO_4_ in the overnight immobilization system led to gradual oxidation of the remaining solution containing CSA. Thus, after the overnight immobilization process, the determination of the remaining yield less represents the main reaction involved due to gradual oxidation. Likewise, a characterization of the column surface by taking out the monolith from the column housing and a cross-section cutting was not feasible. Due to these restrictions, the immobilized column was evaluated through its performance in enantioseparation. Investigation of the immobilization process reproducibility becomes a further step that follows the column evaluation results. This initial report proposed an immobilization method of CSA onto a monolithic column and its performance in enantioseparation.

### 3.2. Enantioseparation

The enantiomeric separation was optimized using phosphate buffer, considering the final solution for the column washing. An acidic pH at 3.5 was applied to achieve fully ionized basic drugs. Hence, the sulfate group of CSA as an anionic chiral selector was predicted to be the most important for enantiorecognition by providing electrostatic interactions with the cationic analyte [38]. Apparently, the addition of organic solvent in the mobile phase progressively decreased the retention time and resulted in poor resolution (data not shown). This typical separation behavior is known in an RP system [32]. However, the presence of an organic solvent in a small amount was found to be required to achieve baseline resolution. Therefore, the mobile phase of 50 mM phosphate buffer pH 3.5 was selected for further study with a small volume addition of organic modifier in the sample solvent. Interestingly, the resolution of the enantiomers was remarkably improved in the presence of MeCN (25% *v*/*v* in water). It might correspond to the higher polarity of MeCN, which elevates enantioselectivity with less competition in hydrogen-bonding interaction compared to MeOH [39]. The immobilized CSA column performed initial enantioseparation on RP mode HPLC. Thus, expansion of the CSA column applicability in NP mode separation remains an interesting aspect of future research.

### 3.3. Molecular Docking and Chromatographic Enantioseparation

HPLC analysis is known to have a limitation in providing direct evidence of the specific binding sites of analytes with the chiral selector. On the other hand, computational docking offers further insights into the detailed geometry information and binding energy of the chiral selector-analyte complex [40]. Thus, computational docking has been proposed as simple approaches for understanding the chiral recognition mechanism on CSPs [41,42,43,44]. A comprehensive docking study on the factors affecting enantioseparation such as solvent effect and other achiral influence was appointed as quantitative approach along with more complex interaction model. However, a molecular docking study was often performed in a vacuum without considering the effect of the medium in the actual separation condition. Consequently, a difference in the predicted energy by molecular modeling and the result from the HPLC analysis might occur. Despite that fact, docking approach delivers a close qualitative estimation regarding the nature of intermolecular forces responsible for the complexes. Although molecular docking does not account for the actual separation conditions, strong correlations of binding affinity scores with chromatography elution orders have been reported [45,46]. In order to assess feasible molecular interactions between the immobilized CSA stationary phase and the analyte, a basic docking approach using AutoDock 4.2.6 was applied.

In this study, the molecular docking was simulated on the three-dimensional structure of CSA and the individual enantiomers at acidic pH. The molecules of CSA and enantiomers were set up as a rigid selector and flexible ligands, respectively. The most stable complexes result in binding energies (Δ*G*) that do not take solvation effects into account. Even though these limitations might lead to the differences between the calculated binding energies and experimental results [29], the applied method offers preliminary insights into enantiomer-specific interactions of the study-related structures [45]. Herein, the sulfate group of CSA was found to play an important role in the chiral recognition of both drug models. It showed a close agreement with the previous study by Nishi et al. using capillary electrophoresis [17]. The difference in binding forces and the Δ*G* values between two enantiomers might define their separation on CSA column chromatography. Based on the predicted binding energies, *R*-(+)-amlodipine and *R*-(+)-verapamil might have retained longer in the column than their counterparts.

## 4. Materials and Methods

### 4.1. Chemicals and Reagents

Methanol and acetonitrile (gradient grade for liquid chromatography) were obtained from Merck (Darmstadt, Germany). Sulfuric acid (H_2_SO_4_) 96%, *ortho*-phosphoric acid (H_3_PO_4_, 85%), sodium periodate (NaIO_4_), sodium cyanoborohydride (NaCNBH_3_), ethylenediamine (NH_2_CH_2_CH_2_NH_2_), disodium hydrogen phosphate (Na_2_HPO_4_), chondroitin sulfate A sodium salt from bovine trachea, (*R*,*S*)-amlodipine as amlodipine besylate, and (*R*,*S*)-verapamil (pharmaceutical secondary standard) were acquired from Sigma-Aldrich Chemie GmbH (Steinheim, Germany). Water was purified by Arium^®^ pro UF/VF-Sartophore 0.22 µm water purification system from Sartorius Weighing Technology GmbH (Göttingen, Germany).

### 4.2. Instrumentation and Chromatographic Conditions

HPLC analysis was conducted using a VWR^TM^-Hitachi (VWR International GmbH, Darmstadt, Germany) consisting of an L-2455 DAD detector, an L-2130 pump, and an L-2200 autosampler. System management and data acquisition were performed by EZChrom Elite^®^ 3.3.2 SP2 Software (VWR International GmBH, Darmstadt, Germany) involving integration parameters of peak area, retention time, and resolution. A Chromolith^®^ Widepore 300 Epoxy 100-4.6 mm HPLC column was kindly provided by Merck KGaA, Germany. Enantiomer separations were performed at ambient column temperature and flow rate of 0.8 mL/min with a mobile phase 50 mM Na_2_HPO_4_ pH 3.5 (adjusted by addition of about 4 mL H_3_PO_4_ 85% in 1 L buffer solution). An injection volume of 20 µL was set up at detection wavelengths of 230 nm and 240 nm.

### 4.3. Preparation of the Immobilized CSP

#### 4.3.1. Conversion of Epoxy Groups and Immobilization of Diamine Spacer

An immobilization approach was applied to develop a CSA-based CSP via a Schiff base formation. A Chromolith^®^ Widepore 300 Epoxy 100-4.6 mm monolithic HPLC column was used as a backbone of the immobilized CSA. Epoxy groups of the monolithic column were first converted to diol groups by hydrolyzation. This step was applied at a flow rate of 0.2 mL/min using 2% *v*/*v* H_2_SO_4_ in water for 24 h. In order to cleave the diol groups to an aldehyde, the process was continued by rinsing the column with 100 mM NaIO_4_ in 20% *v*/*v* MeOH (aqueous solution) at a flow rate of 0.2 mL/min for 15 h. At the following step, 83 mM ethylenediamine in 50 mM Na_2_HPO_4_ containing a mixture of 1.9 M (NH_4_)_2_SO_4_ pH 8.0 and 5 mM NaCNBH_3_ was introduced into the column at a flow rate of 0.2 mL/min for 20 h. The immobilization of diamine-spacer was ended by flushing the column with 20 mM NaCNBH_3_ in 50 mM Na_2_HPO_4_ pH 3.0 at a flow rate of 0.2 mL/min for 8 h, and neutralizing with 100 mM Na_2_HPO_4_ pH 7.4 at a flow rate 0.2 mL/min for 5 h. All of the employed solutions in this step were prepared in water as solvent.

#### 4.3.2. Oxidation of CSA

The oxidation of 1% *w*/*v* CSA was applied by converting the hydroxy groups to the aldehyde groups using 100 mM NaIO_4_ in 20% *v*/*v* MeOH (aqueous solution).

#### 4.3.3. Immobilization of CSA

The immobilization via Schiff base formation was conducted by introducing a mixture of the oxidized CSA (1% *w*/*v*) and 5 mM NaCNBH_3_ as a reductant containing 1.9 M (NH_4_)_2_SO_4_ pH 8.0 in 50 mM Na_2_HPO_4_, at a constant low flow rate of 0.1 mL/min for 20 h. In the following step, solution of 20 mM NaCNBH_3_ in 50 mM Na_2_HPO_4_ pH 3.0 was employed at a flow rate of 0.2 mL/min for 8 h. As the last step, the column was flushed using 50 mM Na_2_HPO_4_ pH 7.4 at 0.2 mL/min for 5 h. 

### 4.4. Preparation of Bulk Samples 

Stock solutions of AML and VER were prepared individually at the concentration of 1.0 mg/mL in 5% *v*/*v* MeOH (aqueous solution). The samples were freshly prepared by diluting each stock solution into 30 µg/mL in various ratios of MeOH as well as acetonitrile (MeCN) as solvent.

### 4.5. Method Validation

Validation of enantioseparation method on the immobilized CSA-based column was performed in the range concentration of AML (50–120 µg/mL) and VER (50–200 µg/mL) at a wavelength of 240 nm and 230 nm, respectively. Phosphate buffer 50 mM at acidic pH 3.5 was chosen at a flow rate 0.8 mL/min. Limit of detection (LOD) and limit of quantitation (LOQ) were calculated as 3.3 times and 10 times the root mean square error (RMSE) divided by the slope of the calibration curve, respectively. Intraday and interday precision of the peak area ratio were determined at three concentrations within 50–100 µg/mL.

### 4.6. Determination of Enantiomers in Commercial Tablets

A further step was taken in order to perform enantioseparation on the immobilized column using commercial tablet matrices of AML (5 mg amlodipine besylate) and VER (80 mg verapamil HCl). Series concentrations of grounded AML and VER tablets were treated individually and dissolved in 5% *v*/*v* MeOH (aqueous solution) by ultrasonication for 5 min at room temperature. Finally, the samples were filtered through a 0.22 µm filter membrane to obtain a clear solution and diluted in 25% *v*/*v* MeCN (aqueous solution) to final concentrations of 60–100 µg/mL of the active substance. Three samples were prepared for each drug and injected in triplicate.

### 4.7. Data Evaluation

Resolution (*R*_s_) between two enantiomers was determined by the mid-height of the peaks, where $t_{1}$ and $t_{2}$ represent the retention times of the first and second eluted peaks, respectively, while $w_{1}$ and $w_{2}$ correspond to widths at the mid-height of the peaks. Retention factors of $k_{1}$ and $k_{2}$ are described as the ratio of Δ*t* between two enantiomers with void value $\left(t_{0}\right)$. *R*s and enantioselectivity (α) were calculated using Equation (1) and Equation (2), respectively.

(1) $$R_{s}=1.18\frac{t_{1}-t_{2}}{w_{1}+w_{2}}$$

(2) $$\alpha =\frac{k_{2}}{k_{1}}$$

### 4.8. Molecular Docking

In general, as a polysaccharide, CSA exposes many types of chiral binding sites. However, the determination is challenging [40]. Thus, a computational method using molecular docking was employed to figure out the feasible complexes. The simulation was conducted using a 3D conformer of the CSA structure taken from the Protein Data Bank (PDB) server entry 4N8W (rcsb.org/structure/4N8W) [20]. The selected CSA consisting of three monomer units was extracted from its crystal structure 4N8W (cathepsin K-chondroitin sulfate complex). Drug structures of *R*-(+)-amlodipine CID_9801597, *S*-(−)-amlodipine CID_9822750, *R*-(+)-verapamil CID_65808, *S*-(−)-verapamil CID_92305 were selected from Pubchem. The structures of the enantiomers were stabilized through MMFF94 geometry optimization using Avogadro version 1.2.0 software and finalized in mol2 format. AutoDock 4.2.6 software (The Scripps Research Institute, La Jolla, CA, USA) was employed to accomplish docking calculations using the Lamarckian Genetic algorithm. In order to involve the charges of drug-related structures, the Gasteiger–Hückel calculation was applied. Grid box size of 30 × 30 × 30 Å was set up with a spacing of 0.375 Å. For each final structure of complexes of 250 independent docking runs was performed. The lowest binding energies represent the most stable analyte-selector complex. Biovia Discovery Studio Visualizer v20.1.0.19295, copyright © 2019 (Dassault Systèmes Biovia Corp, Vélizy-Villacoublay, France) was utilized to visualize the complexes.

## 5. Conclusions

This preliminary study reports the in house generation of an immobilized CSA-based CSP and its performance in chromatographic enantioseparation. CSA immobilization was successfully conducted onto a Chromolith^®^ Widepore 300 Epoxy 100-4.6 mm HPLC column via a reductive amination strategy involving Schiff base formation in the presence of a diamine-spacer. The modified column exhibited chiral separation toward AML and VER in bulk samples and tablet matrices. Molecular docking projected insights into feasible complexes along with the molecular interaction between CSA and the individual enantiomers, which were found to be dominated by hydrogen bonding. The difference of binding energy (Δ*G*) of two enantiomers might correspond to their retention on the CSA-based chromatography column. A study on the immobilized CSA column applicability in various HPLC modes remains attractive for future research.

## Figures and Tables

**Table 1:** Evaluation of CSA column.

Parameter	AML	VER
$t_{1\;}$(min)	2.2 ± 3.3 × 10^−3^	2.2 ± 2.0 × 10^−3^
$t_{2\;}$(min)	3.2 ± 3.7 × 10^−2^	3.6 ± 0.2
*R*_s_	3.2 ± 8.5 × 10^−2^	3.6 ± 8.5 × 10^−2^
$k_{1\;}$	1.4 ± 3.0 × 10^−3^	1.4 ± 3.0 × 10^−3^
$k_{2\;}$	2.5 ± 7.4 × 10^−2^	2.9 ± 0.2
α	1.8 ± 2.8 × 10^−2^	2.1 ± 0.1
$N_{1\;}$	4532 ± 408	4407 ± 368
$N_{2}$	1049 ± 92	589 ± 36

*t*_1_: first eluted enantiomer; *t*_2_: second eluted enantiomer. Separation conditions: mobile phase: 50 mM phosphate buffer pH 3.5; flow rate 0.8 mL/min; sample solvent: 25% *v*/*v* MeCN (in water); injection volume: 20 µL; UV detections: 240 nm (AML) and 230 nm (VER). *N*_1_: number of theoretical plates of first eluted enantiomer; *N*_2_: number of theoretical plates of second eluted enantiomer.

**Table 2:** Validation of the enantioseparation method using immobilized-CSA column.

Parameter	AML		VER	
	$t_{1\;}$	$t_{2\;}$	$t_{1\;}$	$t_{2\;}$
Range (µg/mL)	50–120	50–120	50–200	50–200
Linearity (*R*^2^)	0.9995	0.9985	0.9989	0.9988
Regression	y = 95.3 × 10^3^ x + 132.6 × 10^4^	y = 102.9 × 10^3^ x − 373.7 × 10^4^	y = 90.5 × 10^3^ x + 147.5 × 10^4^	y = 80.8 × 10^3^ x − 102.3 × 10^4^
LOD ^a^ (µg/mL)	2	3	4	6
LOQ ^b^ (µg/mL)	6	10	11	17
Intraday precision *	1.0–1.3%	1.0–1.3%	0.5–1.2%	0.5–1.2%
Interday precision **	1.2–1.8%	1.2–1.8%	1.1–1.2%	1.1–1.2%
Accuracy	99–102%	98–102%	95–102%	98–104%

^a^ based on 3.3 RMSE/slope. ^b^ based on 10 RMSE/slope; RMSE: root mean square error. * repeatability of peak area ratio (*n*: samples = 9; *df*: degrees of freedom = 6). ** repeatability of peak area ratio (days: 3). *t*_1_: first eluted peak. *t*_2_: second eluted peak.

**Table 3:** Proposed interactions by molecular docking and binding energies.

Enantiomer	Type of Interaction	Functional Group	Distance (Å)	Δ*G* ^a^ (kcal/mol)	ΔΔ*G* ^b^ (kcal/mol)
*R*-(+)-amlodipine	Electrostatic *	S-O···N	4.42	−4.84	0.96
	Electrostatic *	-O···N	4.48		
	Hydrogen bond	-OH···O-	2.09		
	Hydrogen bond	-O-···HN	2.13		
	Hydrogen bond	S=O···HN	1.80		
	Hydrogen bond	S=O···HN	2.33		
*S*-(−)-amlodipine	Electrostatic *	S=O···N	3.71	−3.88	
	Hydrogen bond	-O-···HN	2.03		
	Hydrogen bond	S=O···HN	1.61		
	Electrostatic **	S-O···chlorophenyl ring	3.59		
*R*-(+)−verapamil	Hydrogen bond	-O-···HN	2.20	−3.81	0.73
	π-Sulfur	S···dimetoxyphenyl ring	5.92		
	π-Sulfur	S···dimetoxyphenyl ring	5.53		
*S*-(−)-verapamil	Hydrogen bond	S=O···HN	2.35	−3.08	
	Electrostatic *	S-O···N	4.53		
	π-Sulfur	S···dimetoxyphenyl ring	4.48		

^a^ Δ*G*: the lowest docking energy of the best cluster; ^b^ Δ*GG =* |Δ*G*_R_ *−* Δ*G*_S_|; * attractive charge; ** π-anion.

## Data Availability

The data presented in this study are available within the article or on request from the corresponding author.

## References

[1] Chankvetadze B. (2020). Recent trends in preparation, investigation and application of polysaccharide-based chiral stationary phases for separation of enantiomers in high-performance liquid chromatography. Trends Analyt. Chem..

[2] Chankvetadze B., Saito M., Yashima E., Okamoto Y. (1997). Enantioseparation using selected polysaccharides as chiral buffer additives in capillary electrophoresis. J. Chromatogr. A.

[3] Berthod A. (2010). Chiral Recognition in Separation Methods.

[4] Ikai T., Okamoto Y. (2009). Structure control of polysaccharide derivatives for efficient separation of enantiomers by chromatography. Chem. Rev..

[5] Chankvetadze B. (2012). Recent developments on polysaccharide-based chiral stationary phases for liquid-phase separation of enantiomers. J. Chromatogr. A.

[6] Shen J., Ikai T., Okamoto Y. (2014). Synthesis and application of immobilized polysaccharide-based chiral stationary phases for enantioseparation by high-performance liquid chromatography. J. Chromatogr. A.

[7] Niedermeier S., Matarashvili I., Chankvetadze B., Scriba G.K.E. (2018). Simultaneous determination of dextromepromazine and related substances 2-methoxyphenothiazine and levomepromazine sulfoxide in levomepromazine on a cellulose tris(4-methylbenzoate) chiral column. J. Pharm. Biomed. Anal..

[8] Okamoto Y., Aburatani R., Hatada K. (1987). Cellulose tribenzoate derivates as chiral stationary phases for high-performance liquid chromatography. J. Chromatogr..

[9] Francotte E., Huynh D. (2002). Immobilized halogenophenylcarbamate derivatives of cellulose as novel stationary phases for enantioselective drug analysis. J. Chromatogr. A.

[10] Francotte E., Zhang T. (2016). Preparation and evaluation of immobilized 4-methylbenzoylcellulose stationary phases for enantioselective separations. J. Chromatogr. A.

[11] Geryk R., Kalíková K., Vozka J., Plecitá D., Schmid M.G., Tesařová E. (2014). Enantioselective potential of chiral stationary phases based on immobilized polysaccharides in reversed phase mode. J. Chromatogr. A.

[12] Bezhitashvili L., Bardavelidze A., Ordjonikidze T., Chankvetadze L., Chity M., Farkas T., Chankvetadze B. (2017). Effect of pore-size optimization on the performance of polysaccharide-based superficially porous chiral stationary phases for the separation of enantiomers in high-performance liquid chromatography. J. Chromatogr. A.

[13] Bezhitashvili L., Bardavelidze A., Mskhiladze A., Gumustas M., Ozkan S.A., Volonterio A., Farkas T., Chankvetadze B. (2018). Application of cellulose 3,5-dichlorophenylcarbamate covalently immobilized on superficially porous silica for the separation of enantiomers in high-performance liquid chromatography. J. Chromatogr. A.

[14] Minakuchi H., Nakanishi K., Soga N., Ishizuka N., Tanaka N. (1996). Octadecylsilylated porous silica rods as separation media for reversed-phase liquid chromatography. Anal. Chem..

[15] Chankvetadze B., Ikai T., Yamamoto C., Okamoto Y. (2004). High-performance liquid chromatographic enantioseparations on monolithic silica columns containing a covalently attached 3,5-dimethylphenylcarbamate derivative of cellulose. J. Chromatogr. A.

[16] Tsioupi D.A., Stefan-Vanstaden R.-I., Kapnissi-Christodoulou C.P. (2013). Chiral selectors in CE: Recent developments and applications. Electrophoresis.

[17] Nishi H. (1996). Enantiomer separation of basic drugs by capillary electrophoresis using ionic and neutral polysaccharides as chiral selectors. J. Chromatogr. A.

[18] Gotti R., Cavrini V., Andrisano V., Mascellani G. (1998). Dermatan sulfate as useful chiral selector in capillary electrophoresis. J. Chromatogr. A.

[19] Zhang Q., Du Y., Chen J., Xu G., Yu T., Hua X., Zhang J. (2014). Investigation of chondroitin sulfate D and chondroitin sulfate E as novel chiral selectors in capillary electrophoresis. Anal. Bioanal. Chem..

[20] Aguda A.H., Panwar P., Du X., Nguyen N.T., Brayer G.D., Brömme D. (2014). Structural basis of collagen fiber degradation by cathepsin K. Proc. Natl. Acad. Sci. USA.

[21] El Deeb S., Ma B.N., Gust R. (2012). Development and validation of a LC method for the separation and determination of the anticancer-active Fe(III) (4-methoxy-salophene) using the new second-generation monolith. J. Sep. Sci..

[22] Kaminski L., El Deeb S., Wätzig H. (2008). Repeatability of monolithic HPLC columns while using a flow program. J. Sep. Sci..

[23] El Deeb S., Preu L., Wätzig H. (2007). A strategy to develop fast RP-HPLC methods using monolithic silica columns. J. Sep. Sci..

[24] Mallik R., Hage D.S. (2008). High-performance affinity monolith chromatography: Development and evaluation of human serum albumin columns. J. Pharm. Biomed. Anal..

[25] Mallik R., Jiang T., Hage D.S. (2004). Development of an affinity silica monolith containing human serum albumin for chiral separations. Anal. Chem..

[26] Pfaunmiller E.L., Hartmann M., Dupper C.M., Soman S., Hage D.S. (2012). Optimization of human serum albumin monoliths for chiral separations and high-performance affinity chromatography. J. Chromatogr. A.

[27] Pfaunmiller E.L., Paulemond M.L., Dupper C.M., Hage D.S. (2013). Affinity monolith chromatography: A review of principles and recent analytical applications. Anal. Bioanal. Chem..

[28] Gotti R., Fiori J., Calleri E., Temporini C., Lubda D., Massolini G. (2012). Chiral capillary liquid chromatography based on penicillin G acylase immobilized on monolithic epoxy silica column. J. Chromatogr. A.

[29] Li W., Liu C., Tan G., Zhang X., Zhu Z., Chai Y. (2012). Molecular modeling study of chiral separation and recognition mechanism of β-adrenergic antagonists by capillary electrophoresis. Int. J. Mol. Sci..

[30] Zhao Y., Li S., Wang X., Yu J., Song Y., Guo X. (2019). Enantioseparation and molecular modeling study of five β-adrenergic blockers on Chiralpak IC column. Chirality.

[31] Karlsson A., Nystrom A. (2001). Addition of organic modifiers to control retention order of enantiomers of dihydropyridines on chiral-AGP. Anal. Chem..

[32] Jibuti G., Mskhiladze A., Takaishvili N., Karchkhadze M., Chankvetadze L., Farkas T., Chankvetadze B. (2012). HPLC separation of dihydropyridine derivatives enantiomers with emphasis on elution order using polysaccharide-based chiral columns. J. Sep. Sci..

[33] Li M., Zhao Y., Zhou L., Yu J., Wang J., Guo X. (2018). Study of the enantiomeric separation of the anticholinergic drugs on two immobilized polysaccharide-based chiral stationary phases by HPLC and the possible chiral recognition mechanisms. Electrophoresis.

[34] Wolf C. (2005). Stereolabile chiral compounds: Analysis by dynamic chromatography and stopped-flow methods. Chem. Soc. Rev..

[35] Sabia R., Ciogli A., Pierini M., Gasparrini F., Villani C. (2014). Dynamic high performance liquid chromatography on chiral stationary phases. Low temperature separation of the interconverting enantiomers of diazepam, flunitrazepam, prazepam and tetrazepam. J. Chromatogr. A.

[36] He H., Liu Y., Sun C., Wang X., Pham-Huy C. (2004). Effect of temperature on enantiomer separation of oxzepam and lorazepam by high-performance liquid chromatography on a β-cyclodextrin derivatized bonded chiral stationary phase. J. Chromatogr. Sci..

[37] Keiko N.A., Vchislo N.V., Stepanova L.G., Larina L.I. (2008). Condensation of 2-alkoxypropenals with *N*,*N*-and *N*,*O*-1,2-binucleophiles. A route to 2-(1’-alkoxyvinyl)imidazolidines and -oxazolidines. Chem. Heterocycl. Compd..

[38] Tabani H., Mahyari M., Sahragard A., Fakhari A.R., Shaabani A. (2015). Evaluation of sulfated maltodextrin as a novel anionic chiral selector for the enantioseparation of basic chiral drugs by capillary electrophoresis. Electrophoresis.

[39] Amut E., Fu Q., Fang Q., Liu R., Xiao A., Zeng A., Chang C. (2010). In situ polymerization preparation of chiral molecular imprinting polymers monolithic column for amlodipine and its recognition properties study. J. Polym. Res..

[40] Zhu B., Zhao F., Yu J., Wang Z., Song Y., Li Q. (2018). Chiral separation and a molecular modeling study of eight azole antifungals on the cellulose tris(3,5-dichlorophenylcarbamate) chiral stationary phase. N. J. Chem..

[41] Yashima E., Yamada M., Kaida Y., Okamoto Y. (1995). Computational studies on chiral discrimination mechanism of cellulose trisphenylcarbamate. J. Chromatogr. A.

[42] Szabó Z.-I., Tóth G., Völgyi G., Komjáti B., Hancu G., Szente L., Sohajda T., Béni S., Muntean D.-L., Noszál B. (2016). Chiral separation of asenapine enantiomers by capillary electrophoresis and characterization of cyclodextrin complexes by NMR spectroscopy, mass spectrometry and molecular modeling. J. Pharm. Biomed. Anal..

[43] Peluso P., Dessì A., Dallocchio R., Mamane V., Cossu S. (2019). Recent studies of docking and molecular dynamics simulation for liquid-phase enantioseparations. Electrophoresis.

[44] Sardella R., Camaioni E., Macchiarulo A., Gioiello A., Marinozzi M., Carotti A. (2020). Computational studies in enantioselective liquid chromatography: Forty years of evolution in docking- and molecular dynamics-based simulations. Trends Anal. Chem..

[45] Ali I., Suhail M., Asnin L. (2018). Chiral separation and modeling of quinolones on teicoplanin macrocyclic glycopeptide antibiotics CSP. Chirality.

[46] Raikar P., Gurupadayya B., Mandal S.P., Narhari R., Subramanyam S., Srinivasu G., Rajan S., Saikumar M., Koganti S. (2020). Bioanalytical chiral chromatographic technique and docking studies for enantioselective separation of meclizine hydrochloride: Application to pharmacokinetic study in rabbits. Chirality.

